# Mapping the Dynamics of Contemporary PRRSV-2 Evolution and Its Emergence and Spreading Hotspots in the U.S. Using Phylogeography

**DOI:** 10.3390/pathogens12050740

**Published:** 2023-05-20

**Authors:** Nakarin Pamornchainavakul, Igor A. D. Paploski, Dennis N. Makau, Mariana Kikuti, Albert Rovira, Samantha Lycett, Cesar A. Corzo, Kimberly VanderWaal

**Affiliations:** 1 Department of Veterinary Population Medicine, University of Minnesota, St. Paul, MN 55108, USA; pamor001@umn.edu (N.P.); ipaplosk@umn.edu (I.A.D.P.); dmakau@umn.edu (D.N.M.); mkikuti@umn.edu (M.K.); rove0010@umn.edu (A.R.); corzo@umn.edu (C.A.C.); 2 Veterinary Diagnostic Laboratory, University of Minnesota, St. Paul, MN 55108, USA; 3 Roslin Institute, University of Edinburgh, Edinburgh EH25 9RG, UK; samantha.lycett@ed.ac.uk

**Keywords:** porcine reproductive and respiratory syndrome virus, phylogeography, molecular epidemiology, virus evolution, disease emergence, subsampling

## Abstract

The repeated emergence of new genetic variants of PRRSV-2, the virus that causes porcine reproductive and respiratory syndrome (PRRS), reflects its rapid evolution and the failure of previous control efforts. Understanding spatiotemporal heterogeneity in variant emergence and spread is critical for future outbreak prevention. Here, we investigate how the pace of evolution varies across time and space, identify the origins of sub-lineage emergence, and map the patterns of the inter-regional spread of PRRSV-2 Lineage 1 (L1)—the current dominant lineage in the U.S. We performed comparative phylogeographic analyses on subsets of 19,395 viral ORF5 sequences collected across the U.S. and Canada between 1991 and 2021. The discrete trait analysis of multiple spatiotemporally stratified sampled sets (*n* = 500 each) was used to infer the ancestral geographic region and dispersion of each sub-lineage. The robustness of the results was compared to that of other modeling methods and subsampling strategies. Generally, the spatial spread and population dynamics varied across sub-lineages, time, and space. The Upper Midwest was a main spreading hotspot for multiple sub-lineages, e.g., L1C and L1F, though one of the most recent emergence events (L1A(2)) spread outwards from the east. An understanding of historical patterns of emergence and spread can be used to strategize disease control and the containment of emerging variants.

## 1. Introduction

Porcine reproductive and respiratory syndrome (PRRS) is considered to be one of the most significant pig diseases in the U.S. and worldwide [1]. The predominant virus species that causes this disease in the U.S. is porcine reproductive and respiratory syndrome virus type 2 (PRRSV-2) [2,3], an RNA virus belonging to the family *Arteriviridae* in the *Nidovirales* order [4]. PRRSV-2 interrupts the function of the innate immune system [5], weakens adaptive immunity [6], induces cell death [7,8], and triggers inflammatory responses [9,10,11] in infected pigs, leading to various clinical presentations including late-term abortion and premature farrowing in sows, high mortality in piglets, and failure to thrive in growing pigs [12]. These clinical impacts during PRRS outbreaks directly diminish gross pig production and income on the level of individual farms [13] and at national scales [14,15].

PRRS first appeared in the U.S. in the states of North Carolina, Minnesota, and Iowa in 1987–1988 [16]. After more than 3 decades, 20–30% of sow farms throughout the U.S. pig industry still experience PRRSV-2 outbreaks each year [17]. The persistence of PRRSV-2 is characterized by the cyclical emergence of new genetic variants of the virus [18], the spread of which is then facilitated by the continuous movement of pigs between herds as part of multi-site, vertically integrated production systems. New variants typically emerge in a particular geographic area and then disseminate widely in the industry through routine animal movements [19,20,21,22]. The location of the emergence and the subsequent patterns of spread constitute crucial information, determining where prevention measures should be strengthened to limit pathogen dispersal. Phylogeography, which utilizes evolutionary relationships between viral genetic sequences to reconstruct ancestral locations and migrations, can be a useful tool to address these questions.

PRRSV-2 diversity has conventionally been quantified through variation in the open reading frame 5 (ORF5) gene [2,3,23]. This 603-nucleotide gene encodes the major envelope glycoprotein, in which several immune epitopes reside [24]. Its immunogenic importance, coupled with its high genetic variability between viruses [3,25,26], makes ORF5 a good marker for PRRSV-2 genetic diversity. Historical analysis of PRRSV-2 ORF5 sequences in the U.S. has shown that the PRRSV epidemiological landscape is characterized by the co-existence of multiple groups of genetically distinct viruses [3], referred to as phylogenetic lineages [3,18]. Pairwise genetic distances are typically <11% for sequences within the same lineage, typically with >10% divergence between lineages [18]. These lineages were originally defined by Shi et al., using data from up to 2009 [26]. Since then, PRRSV-2 lineage 1 (L1) has increased from <40% to >60% of sequenced viruses, while other PRRSV-2 lineages have been decreasing over time [3,27]. It is worth noting that the majority of non-L1 viruses detected through sequencing in the past decade (20–30% of sequenced viruses) are L5 and L8 [27], which have been widely used as commercial live-attenuated vaccines, whereas wild-type non-lineage 1 sequences account for <1% of sequences [27]. In addition, lineage 1 has diverged into several subpopulations, i.e., sub-lineages L1A to L1H (typical pairwise genetic distances <8.5% for sequences belonging to the same sub-lineage, and >9% divergence between sub-lineages [18]. The prevalence of different sub-lineages varies geographically and temporally, with cyclic population expansions and contractions of particular groups of viruses being continuously observed [18].

Although patterns of L1 sub-lineage emergence and turnover are well described [3,18], the geographic origin and spreading hotspots of contemporary L1 viruses have not been determined. Such information is crucial for PRRS prevention, management, and containment. The most recent analysis of PRRSV-2 phylogeography in the U.S. used sequences up to 2011 [28], prior to when L1 became the dominant lineage in the U.S. Thus, this earlier work might not be representative of the current epidemiologic situation. To fill these gaps, our objective is to provide an updated analysis of the spatiotemporal dynamics of the spread and population growth of PRRSV-2 L1 over the last three decades in the U.S., with a particular emphasis on understanding the spatiotemporal dynamics that underpin the continued diversification of L1 into numerous sub-lineages. We inferred the phylodynamics of each L1 sub-lineage and identified potential selection pressures associated with phylogenetic divergence that may help to explain the overall L1 phylogeography. Given the large size of the aggregated U.S. PRRSV ORF5 sequence dataset, we also evaluated the robustness of our results with reference to various sub-sampling strategies that aimed to generate spatial-temporally representative subsets for analysis. This study advances our understanding of the large-scale geographic expansions and evolutionary dynamics of the virus; it may help us to determine why PRRSV-2 persistently circulates within the U.S.

## 2. Materials and Methods

### 2.1. Data Sources

Three data sources, the National Center for Biotechnology Information GenBank (NCBI, 1991–2021), the University of Minnesota Veterinary Diagnostic Laboratory (UMN VDL, 2004–2021), and the Morrison Swine Health Monitoring Project (MSHMP, 2010–2021), were accessed in October 2021 to gather PRRSV-2 ORF5 genetic sequences collected in the U.S. Briefly, the UMN VDL has generated virus sequences from throughout the U.S. as part of services rendered to primarily U.S.-based industry clients. MSHMP is a voluntary program established in 2011 that archives a variety of swine disease data from over 35 participating production systems, capturing data from more than 50% of the U.S. breeding population [29]. Outbreaks identified and reported to MSHMP may have an accompanying ORF5 gene sequence. Sequences reported to MSHMP are typically produced by the VDLs of the University of Minnesota, Iowa State University, South Dakota State University, or Kansas State University. All available sequences from Canada (only found in the NCBI GenBank) were also included, given that the routine transport of hogs between the U.S. and Canada [30] might lead to transboundary PRRSV transmission.

The inclusion criteria for sequences were the completeness of the sequence (>580 nucleotides) and the availability of a sample collection date and location (i.e., the state in the U.S. where a sample was collected or, in the case of the UMN VDL, the state where the client who submitted the sample was located). Primary deduplication by sequence identification number was performed between the MSHMP and the UMN VDL datasets. Subsequently, the aggregated dataset of 40,365 ORF5 sequences were aligned using pairwise local alignment in MAFFT v.7.310 [31], and the alignment was used to build an approximately maximum-likelihood tree using FastTree v.2.1.10 [32]. PRRSV-1 sequences detected in the tree were excluded before realigning only the PRRSV-2 ORF5 sequences onto a PRRSV-2 reference (GenBank accession no. NC_038291.1) using pairwise codon alignment via VIRULIGN [33]. Repeated sequences from localized outbreaks caused by highly related viruses (defined by same exact collection date, collected within the same state, and with 100% nucleotide similarity) were deduplicated. After filtering out sequences containing ambiguous nucleotides (*n* = 4644), gaps (*n* = 530), or with signals of potential recombination (*n* = 4) consistently detected by all seven methods implemented in in RDP5 [34], wherein a fully exploratory (all sequences compared to all others) recombination analysis was performed using the RDP [35], GENECONV [36], MaxChi [37], BootScan [38], SiScan [39], Chimaera [40], and 3Seq [41] methods, the curated 29,554 PRRSV-2 ORF5 sequences were classified into (sub-)lineages by measuring the pairwise distance between a sequence to each (sub-)lineage’s anchors, as described elsewhere (Paploski, et al.) [3,18]. Non-L1 sequences were excluded from further analysis. Ultimately, an alignment of just 19,395 PRRSV-2 L1 ORF5 sequences was used for further analyses (Supplementary Figure S1).

### 2.2. Subsampling

**Figure 1:**
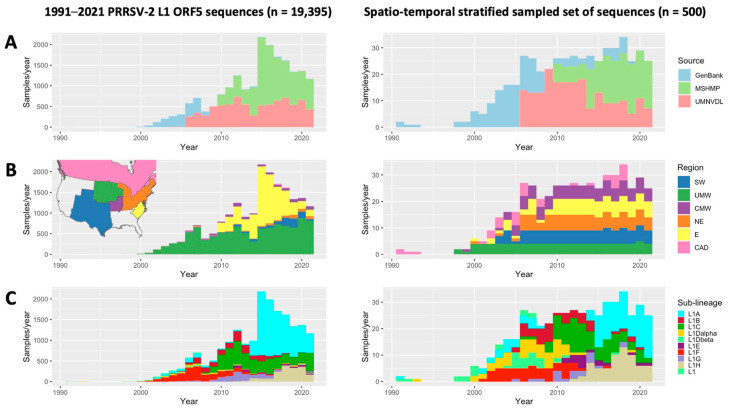
Temporal distribution of the full PRRSV-2 L1 dataset (**left**) and an example of a spatiotemporally stratified subsampled dataset (**right**) colored according to (**A**) source, (**B**) sampling location (region), and (**C**) pre-determined sub-lineage.

The geographic regions utilized for discrete-space phylogeography were adapted from the Swine Health Information Center’s (SHIC) regions, with boundaries creating divisions between major pork-producing areas in the U.S. Due to the high number of available sequences, SHIC’s region 3 (Midwest) was subdivided into 2 regions according to the spatial distribution of the pig population [42,43]. These regions included the Southwest (SW, *n* = 1057 sequences), the Upper Midwest (UMW, *n* = 10,384 sequences), the Central Midwest (CMW, *n* = 827 sequences), the Northeast (NE, *n* = 473 sequences), the East (E, *n* = 6554 sequences), and Canada (CAD, *n* = 80 sequences) (Supplementary Table S1). The Western (W, *n* = 12 sequences) and Southeastern (SE, *n* = 8 sequences) U.S. were not included in the analysis since less than 20 sequences were available from those regions, likely reflecting low pig populations [43]. Given that one of the data sources is a Minnesota-based diagnostic lab, data availability was highly biased towards the Upper Midwest. Imbalances in the number of sequences per region can create analytical biases that influence ancestral state reconstructions produced by phylogeographic models, wherein the model is falsely confident that the overrepresented region is the ancestral region. To diminish this bias and for computational feasibility, the L1 alignment (*n* = 19,395) was sampled 5 times (500 sequences per subset) using a spatiotemporally stratified uniform sampling method with year and region strata. This approach aims to equalize the number of samples included from each region/year; as such, an equal number of sequences were drawn from the available sequences per region for each calendar year from 1991 to 2021, except for some years, in which a few (<5) or no sequences were available from particular regions (Figure 1). Using this approach, the median number of sequences/region/year was 3 (IQR = 0–4) and the total unique sequences utilized across the 5 random data sets was 1765, or 9.1% of all L1 sequences.

As there is some uncertainty about the most appropriate sub-sampling strategy for addressing sampling bias [44,45], two other subsampling approaches were also assessed five times each. For uniform spatial stratified sampling, sub-sampling was performed as described above but ignoring the year. For proportionate stratified sampling, the number of sequences included per region was set to be proportionate to the relative pig population in each region and state, as reported in the 2017 agricultural census [43]. A comparison of the spatiotemporal representations of all three subsampling approaches is shown in the Supplementary Data. (Supplementary Figures S3 and S4)

### 2.3. Phylogeographic Analyses

The origin and frequency of inter-regional spread of PRRSV-2 Lineage 1 and its sub-lineages were estimated from the subsampled sequences via Bayesian discrete phylogeography, which is also referred to as discrete trait analysis (DTA). This analysis treats the sampling location as a discrete trait and then estimates the ancestral locations and geographic migrations of the virus from the trait transitions across the viral evolutionary tree through continuous-time Markov chain (CTMC) modeling [46]. To achieve this, time-scaled phylogenetic trees with phylogeographic inference were reconstructed from each subsampled set using BEAST v.1.10.4 [47]. The models used for the analysis were as follows: the general time reversible with gamma plus invariant site heterogeneity (GTR + I + G) as the nucleotide substitution model, the uncorrelated relaxed clock [48] with log-normal distribution as the molecular clock model, and the non-reversible CTMC for the asymmetric discrete trait substitution model [46]. We additionally inferred the viral population dynamics by specifying the Bayesian Skygrid’s Gaussian Markov random field (GMRF) model [49] as a coalescent. Prior to the Bayesian analysis, the temporal signals of each subsampled set were checked using TempEst v.1.5.3 [50] by analyzing the root-to-tip distances in the maximum likelihood trees built by IQ-TREE [51]. Bayesian analyses were run with 300 million Markov chain Monte Carlo (MCMC)’s chain length. The first 10% of the samples from the MCMC chain were discarded as burn-ins, and the remaining trees were summarized as a maximum clade credibility (MCC) tree via TreeAnnotator v.1.10.4 [52] and visualized using the Nextstrain [53] platform. Phylogeographic and population dynamic outputs were visualized using the ggplot2 [54] package in R [55]. All of these approaches were repeated for each set of sequences belonging to each of the seven sub-lineages identified in the MCC trees, including L1A, L1A(2) (the secondary re-emergence of L1A viruses [18]), L1BG (a monophyletic clade comprising L1B and L1G), L1C, L1E, L1F, and L1H. In total, 50 DTA runs (15 runs from 3 subsampling strategies performed on L1 overall, plus 35 runs from the sub-lineage analyses, for which we only used spatiotemporal subsampling) were performed to infer the spatial-temporal dynamics of the virus. The key phylogeographic results were combined from 5 runs, e.g., the range of median times to the most recent common ancestor (tMRCA), the range of the 95% highest posterior densities (HPDs), and the range of probabilities in the ancestral region.

Maximum likelihood discrete-space phylogeography [56] and Bayesian structured coalescent approximation [57] were explored as alternative analytical approaches and were used to evaluate the sensitivity of our results to the utilized modeling platform. The first method, maximum-likelihood phylogeography, essentially infers ancestral traits across an evolutionary tree’s internal nodes by treating migration between discrete locations in the same way as genetic mutation, given a time-reversible model [56]; it is a non-Bayesian version of the DTA described above. Bayesian structured coalescent approximation extends the Bayesian coalescent model to allow for migration between subpopulations within a structured population and approximates the internal nodes’ trait probability [57,58]. The same five spatiotemporal stratified sampled sets used for DTA were reanalyzed using these methods via TreeTime v.0.8.5 [56] in the Nextstrain’s augur v.10.1.1 pipeline [53] (the maximum-likelihood approach), and the marginal approximation of the structured coalescent (MASCOT) v.2.1.2 [58] package in BEAST v.2.5.1 [59], respectively. Since TreeTime does not require significant amounts of computational power to run a large dataset, the full set of PRRSV-2 L1 ORF5 sequences (*n* = 19,395) was also analyzed by TreeTime. Detailed parameter settings are displayed in the Supplementary Data (Supplementary Table S2). Ancestral locations and their transitions through time from all phylogeographic analyses were summarized using the Babel v.0.4.0 package in BEAST v.2.5.1 [59].

### 2.4. Selective Pressure Analysis

Possible selective pressures throughout PRRSV-2 L1’s evolutionary history were identified using the branch-site test of positive selection [60]. We used a branch-site test because branches can be tied to an inferred geographic region, and we wanted to test the hypothesis that selection pressures vary between different regions. The analysis was performed on each of the five spatiotemporally subsampled MCC trees from DTA and their original sequence alignment. Implemented in aBSREL (adaptive branch-site random effects likelihood) v.2.3 software, all tree branches were tested using an exploratory analysis, in which the optimal ω (non-synonymous to synonymous substitutions ratio or dN/dS) of each branch was inferred by the small-sample Akaike Information Criterion (AIC_c_) and then compared to the null model (ω ≤ 1) using the likelihood ratio test (LRT) [61]. If a branch has the inferred ω > 1 and LRT *p*-value < 0.05, the virus is estimated to evolve under episodic positive (diversifying) selection at that branch.

## 3. Results

### 3.1. Origin of PRRSV-2 L1 and Its Sub-Lineages

**Figure 2:**
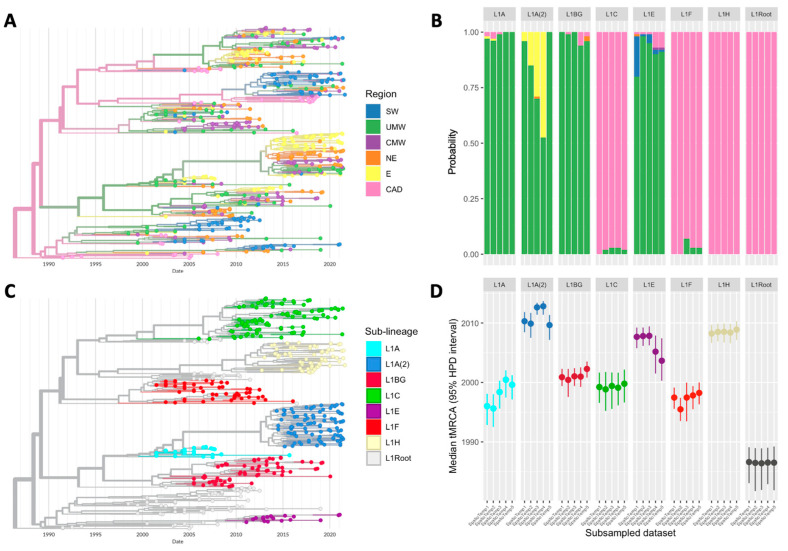
Key results from the DTA of spatiotemporally stratified sampled sets. (**A**) A time-scaled phylogenetic tree of one subsampled set with the tips colored according to the sampling region and the internal branches colored according to the inferred ancestral region. (**B**) Probability (0–1) of region of origin for each L1 sub-lineage and overall L1 from all runs. (**C**) The same timed-scaled tree with tips colored according to the classified sub-lineage. (**D**) Median tMRCA with a 95% HPD interval of L1 and its sub-lineages from the same runs.

The results from five DTAs on different spatiotemporally stratified subsampled sets (*n* = 500 each) suggest that the PRRSV-2 L1 in the U.S. originated in Canada (100% posterior probability) during the late 1980s (median time to the most recent common ancestor (tMRCA) = 1986; (range of 95% highest posterior densities (HPDs) = 1981–1989)). Although the L1A and L1F sub-lineages concurrently diverged from the primitive L1 in the late 1990s (range of median L1A tMRCA = 1995–2000; (range of 95% HPDs = 1992–2002) and range of median L1F tMRCA = 1995–1998; (range of 95% HPDs = 1993–2000)), L1F was likely imported from Canada (93–100% probability), while L1A emerged within the Upper Midwestern U.S. (97–100% probability). Within a few years, two additional sub-lineages, L1C and L1BG, diverged (range of median L1C tMRCA = 1998–1999; (range of 95% HPDs = 1995–2002) and range of median L1BG tMRCA = 2000–2002; (range of 95% HPDs = 1997–2003)). L1C was a sister clade of L1, and L1BG was a sister of L1A on every MCC tree. Like their sisters, L1C and L1BG potentially originated in Canada (97–100% probability) and the Upper Midwest (93–100% probability), respectively. L1 further diverged into another two sub-lineages, L1E and L1H, in the 2000s. L1E was a relatively small clade, directly arising from the basal L1 around the mid-to-late 2000s (range of median L1E tMRCA = 2003–2007; (range of 95% HPDs = 2000–2009)) and was estimated to have emerged in the Upper Midwest (80–98% probability). On the contrary, L1H was a recent sister of L1C and L1F, and its approximated time and place of origin were the late 2000s (range of median L1H tMRCA = 2008; (range of 95% HPDs = 2006–2010)) in Canada (100% probability). The second and larger wave of L1A (L1A(2)) branched out from the original L1A in early 2010s (range medians L1A(2) tMRCA = 2009–2012; (range of 95% HPDs = 2007–2013)). Although the most likely origin of this emergence appears to be the Upper Midwest (52–99% probability across analyses based on 5 subsets of data), the probability that the source was the Eastern U.S. (4–47% probability) could not be ruled out (Figure 2).

When comparing the results generated by the different subsampling techniques, the estimations of both the time and location of origin from the spatially stratified subsampled sets were markedly similar to those from the spatiotemporally stratified subsampling. Unlike the first two techniques, estimations based on the proportionate stratified samples indicate that the L1 virus and all its sub-lineages likely originated in the Upper Midwestern U.S., the region with the proportionately largest pig population and thus the most sequences included in the analysis (48% of the sequences were from the Upper Midwest). Inferred tMRCAs from the population-based subsampling were less consistent compared to the spatially based subsamples (Supplementary Figure S5). 

The virus phylogeography inferred by the DTA differed when using other modeling approaches, despite the same inputs being used (i.e., all models utilized the same subsets produced from spatiotemporally stratified sub-sampling). For instance, the origins of L1BG, L1E, and L1A(2) were more uncertain in the maximum-likelihood-based TreeTime analysis than in DTA; in some cases, three regions were considered almost equally likely to be the putative origin (sub-lineages L1A(2) and L1BG), or different runs produced different results (sub-lineage L1E). That said, the estimations from TreeTime were far more similar to the DTA than the results from MASCOT. In the latter, there were high levels of uncertainty regarding the region of origin for most sub-lineages, and the Eastern U.S. was frequently inferred to be a potential origin of both L1 overall and most sub-lineages. However, DTA, TreeTime, and MASCOT similarly estimated that the origin of L1H was in Canada (100% probability). Focusing on the time of emergence, estimates of tMRCAs and inferred nucleotide substitution rates were similar across all methods (Supplementary Figure S6).

**Figure 3:**
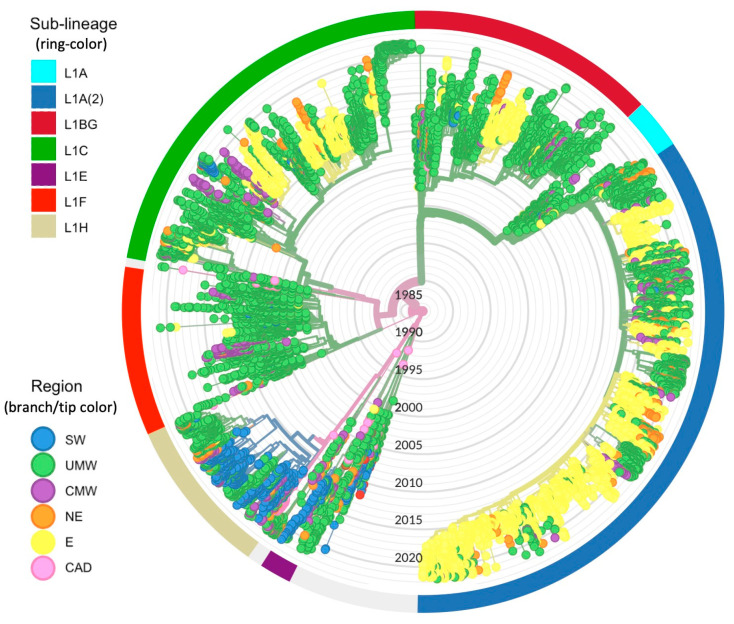
Maximum-likelihood time-scaled phylogenetic tree of the full L1 dataset (*n* = 19,395) estimated by TreeTime. Tips and branches are colored according to the sampling region and inferred ancestral region, respectively. The exterior ring is colored according to L1 sub-lineages based on phylogenetic grouping.

Both the geographic region and the time of origin of the L1 virus estimated from the non-subsampled set (*n* = 19,395) using TreeTime corresponded well to the inference from the spatiotemporally stratified samples subjected to DTA. The only disagreement was the origin of the sub-lineage L1F; the ancestral location of the full dataset was inferred to be the Upper Midwest instead of Canada, possibly as a result of the overrepresentation of the Upper Midwest in this dataset (Figure 3 and Supplementary Figure S7).

### 3.2. Inter-Regional Spread and Spreading Hotspots

**Figure 4:**
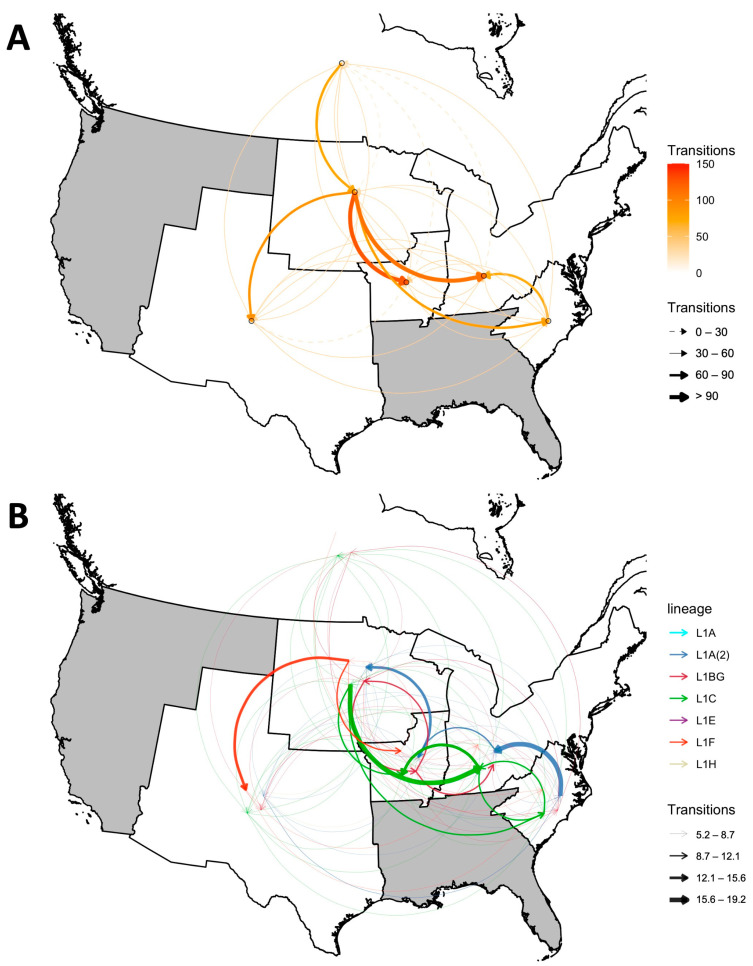
Inter-regional spread of PRRSV-2 L1 in the U.S. estimated by the DTA of spatiotemporally stratified sampled sets. (**A**) Median between-region transitions of the L1 lineage overall. The shade and thickness of the arrows represent the estimated number of transitions. (**B**) Median between-region transitions of each L1 sub-lineage. The color of the arrow represents the sub-lineage, whereas the thickness represents the number of transitions.

The source regions contributing to frequent inter-regional spread (based on the median number of transitions between regions across the five runs) inferred by the phylogeographic analysis were considered hotspots for inter-regional spread. DTA analysis of the spatiotemporally stratified samples suggests that the Upper Midwestern U.S. was the main hotspot for inter-regional spread and was the origin of frequent dissemination events to every region except for Canada. The common destinations of such events were the Central Midwest (~126 transitions since L1 emergence until 2021) and the Northeast (~111 transitions). Canada and the Eastern U.S. can also be considered hotspots, although they mainly spread the virus to only a single adjacent region, such as the Upper Midwest (~75 transitions) and the Northeast (~71 transitions), respectively (Figure 4A). Summarizing the proportion of branches inferred to exist in each region across time (Supplementary Figure S11), L1 was primarily circulating in Canada in the 1990s and was likely introduced to the U.S. through the Upper Midwest during the early 2000s. After around 2005, Canada gradually ceased to be a major player in U.S. inter-regional spread (although we have relatively few Canadian sequences in later years, preventing us from fully understanding its role in later periods). These phylogeographic patterns were relatively stable across different phylogeographic approaches and subsampling techniques, albeit with some variations. For instance, MASCOT approximated that the virus spread from the East to both the Northeast and the Upper Midwest, and that there was no apparent flow from Canada to the U.S., which was inconsistent with the patterns inferred by the other approaches (Supplementary Figures S8 and S9).

At the sub-lineage level, the direction and extent of inter-regional spread were unique for each viral subpopulation. L1C and L1A(2) were the predominant sub-lineages with the highest number of samples across all subsampled datasets (Figure 1, Figure 2 and Figure 3)**.** Nevertheless, the inferred patterns of inter-regional spread moved in opposite directions. The DTA phylogeography estimated that the Upper Midwest was a spreading hotspot for L1C, which disseminated the virus mainly to the Northeast, Central Midwest, and East. L1C was also found to spread in the opposite direction, from the East to the Northeast to the Central Midwest. In contrast, L1A(2) displayed a more unidirectional northwesterly flow from the East to the Northeast to the Central Midwest to the Upper Midwest. With a smaller number of inferred transitions events due to its smaller population size, L1BG mostly circulated back and forth between two Midwestern regions, with some spillover to the Northeast, whereas L1F largely spread from the Upper Midwest to the Southwest, with some introductions to the Central Midwest (Figure 4B)**.** Even though the extent of L1H’s spread was not comparable to the spread of other sub-lineages, all of the phylogenetic trees show that the L1H viruses circulated primarily within the Southwestern U.S., which was also its spreading hotspot according to the DTA inference. The smallest sub-lineages were L1A and L1E, for which no distinct patterns of inter-regional spread were discerned (Supplementary Figure S10)

### 3.3. Population Dynamics, Mutation, and Selection Pressure

**Figure 5:**
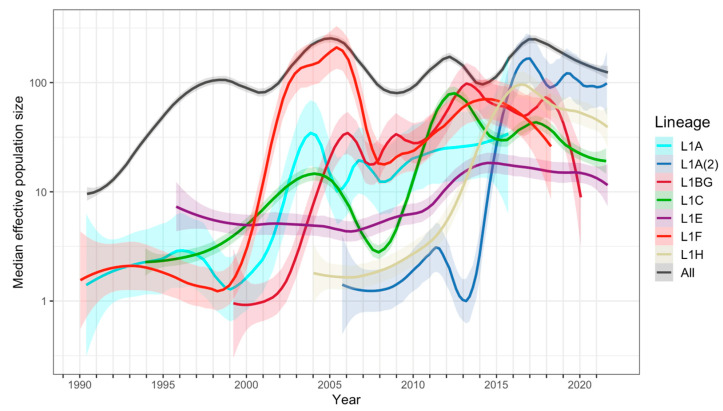
PRRSV-2 L1 population dynamics in the U.S. estimated by Bayesian Skygrid analyses on spatiotemporally stratified sampled sets. Lines with shaded bands are LOESS smoothing curves, with a 95% confidence interval, of the median log effective population sizes from 5 runs of each sub-lineage and overall L1. Lines are colored according to the L1 sub-lineage.

PRRSV-2 L1 population dynamics in the U.S. were summarized from the median effective population sizes inferred by the Bayesian Skygrid analysis on different spatiotemporally stratified sets. The virus population rapidly grew within the first decade (1990–1998), after which the population dynamics were characterized by a wave-like pattern, wherein the effective population size slightly decreased and then increased to a new peak approximately every ~6 years until the present (Figure 5). Over 30 years, there were 4 peaks in the effective population size of L1, each of which was driven by different sub-lineages. The first L1 peak (1998) occurred prior to the peaks of any of the analyzed sub-lineages, and may have resulted from older sub-lineages, such as L1D, which were classified as primitive L1 in this study [18]. Subsequent peaks coincided with the epidemic-like wave of L1F, which appears to have pushed the overall L1 population to the second peak in 2005. The third wave, peaking in 2012, involved L1F, L1BG, and L1C, but increases associated with this peak appear to be most strongly driven by a rapid increase in L1C. The most recent peak (2017) was mainly driven by the sub-lineages L1A(2) and L1H, which emerged in the late 2000s but did not experience rapid growth until the mid 2010s (Figure 5)**.** The Skygrid analyses of the inferred population sizes were remarkedly consistent across the different subsampling techniques, nearly completely overlaying on top of each other, except during the early time period (before 2000), when the size of the first peak in population size varied according to the subsampling strategy (Supplementary Figure S13).

The median nucleotide substitution rate for overall L1, which was computed from the spatiotemporal MCC trees, was 8.6 × 10^−3^ (IQR = 7.7 × 10^−3^ − 1.0 × 10^−2^) substitutions per nucleotide site per year (s/n/y). There was no significant difference between sub-lineages or geographic regions, nor was there a clear temporal pattern in branch-specific substitution rates (Supplementary Figures S14 and S15).

The selective pressure analysis of the spatiotemporal stratified sequences and trees estimated that ORF5 mostly evolved under near-neutrality, regardless of lineage or region (median and mode ω = 1, IQR = 0.38 − 2.11). Although the adaptive branch-site model’s ω showed that the ancestral viruses of several trees’ branches also evolved under both extremely high-purifying and positive selection, only three branches from three different subsampled trees can be considered to be positively selected (*p*-value < 0.05) according to the likelihood ratio test (LRT). These rare episodic positive selection events displayed no clear spatial or temporal patterns. One was identified at an internal branch of primitive L1 in 1992, prior to the virus being introduced from Canada; another was a terminal branch of primitive L1 in 2000 in the East, which originated from Canada, and a third was a terminal branch of sub-lineage L1C in 2014 in the East, without any associated inter-regional transition. The positive selected branches did not have higher evolutionary rates compared to the others (Supplementary Figures S16 and S17).

## 4. Discussion

In this work, we aggregated several large-scale PRRSV-2 L1 ORF5 sequence datasets from the U.S. and Canada to reconstruct the historical patterns of lineage emergence, inter-regional spread, and population dynamics using phylogeography. Using such a large dataset allowed us to apply different subsampling techniques and phylogeographic approaches and test the sensitivity of our inferences to the sub-sampling technique and modeling approach utilized [44,62]. We found evidence that L1 viruses overall, as well as the sub-lineages L1C, L1F, and L1H, potentially originated in Canada, while the sub-lineages L1A, L1A(2), L1BG, and L1E emerged within the Upper Midwestern U.S. However, the initial region of origin might not always be the hotspot that is most responsible for frequent inter-regional spread. Moreover, the hotspot and spreading patterns of each viral subpopulation varied. For example, the opposing directionality of the dissemination of the two currently predominant sub-lineages, L1C and L1A(2), demonstrated the varied phylogeographic histories of different subpopulations. This complexity was also found in the temporal demographic inference, which shows that the contribution of each co-existing sub-lineage to the overall L1 population dynamics has fluctuated through time.

The majority of our inferences, as well as a previous phylogeographic analysis published in 2013 [28], indicate that L1 viruses in the U.S. originated in Canada. This does not seem to be due to lack of sequence data in the U.S. before 1998 (the earliest detected L1 sequence in the U.S.) [63], given that there are abundant sequence data available (>230 sequences, all non-L1) from throughout the U.S. since 1989 [23,64,65,66,67]. The detection of L1 sequences as early as 1991 in Canada, but not in the U.S., [68,69,70,71] further supports the conclusions of our phylogeographic analysis. Many of the early Canadian sequences were not included in the analysis where sub-sampling was proportional to pig population size; the preponderance of Upper Midwestern sequences and the exclusion of early Canadian sequences in those sub-samples resulted in the Upper Midwestern U.S. being the inferred origin of L1 and all of its sub-lineages, but we believe this to be an analytical artefact of the overrepresentation of this region. Thus, we contend that spatiotemporally stratified sub-sampling was the best approach to overcome the sampling biases in our dataset.

Performing the analysis with the spatiotemporal sub-sampled sequences in MASCOT, which uses a forwards/backwards algorithm [72] with a structured coalescent model [57], resulted in drastically different results, in which all L1 viruses except L1H were inferred to originate in the East. Such a pattern would be possible only if the L1 virus did truly exist in the East undetected during or before the 1990s but had never been sampled or sequenced. In fact, according to our data, at least 40 samples from the Eastern region were sequenced between 1992 and 1999, but none of them was classified as L1. This further supports our conclusion that conducting DTA of the spatiotemporally stratified samples was the most reliable and robust approach for our data.

While the Canadian origin of the L1 lineage is well supported, the evidence for Canada’s potential role in subsequent inter-regional spread was inconclusive, given the available dataset. The introductions of the primitive L1, L1F (circa 1998–2001), L1C (circa 2000–2005), and L2H (circa 2009–2011) from Canada to the U.S. were highlighted, while reverse dissemination from the U.S. back to Canada was rare. This unidirectional pattern is not surprising, given that the U.S. imports millions of feeder and finishing pigs per year from Canada but exports only a few thousand pigs back to Canada annually [30]. The changes in the structures of the U.S. and Canada’s pig industries that led to this phenomenon have been described elsewhere [73,74], but it is worth noting that 90% of imported pigs are moved into the Upper Midwest [75]. L1H, the most recent sub-lineage originating from Canada, appears to have been introduced to the U.S. in the late 2000s when the Canada-to-U.S. pig imports were at their peak (8 to 10 million heads/year) [30]. Once the virus has been introduced to the U.S., the Upper Midwest appears to be the epicenter of inter-regional spread; this is likely because it is a hub for interstate pig shipments, given the number of harvest plants and the proximity to corn and soybean crops that are used in feed [76,77]. Notably, this general pattern of virus movement from Canada to the Upper Midwest, following by inter-regional spread within the U.S., mirrors the phenomenon observed for swine influenza [78,79]. 

The question of whether Canada continues to disseminate viruses into the U.S. cannot be fully answered due the reduced number of Canadian L1 ORF5 sequences available in the NCBI GenBank database in recent decades. Reductions in the number of PRRSV-2 sequences available in GenBank also occurred in the U.S. After 2010; when sequencing technology became more accessible and affordable, more ORF5 sequencing was likely requested by field veterinarians for diagnostic purposes, which could explain the reduced number of publicly available sequences. Thus, data sharing via GenBank, especially for the kind of sensitive metadata that we needed, i.e., sampling dates and locations (states), has been decreasing due to confidentiality concerns.

Phylogeographic and phylodynamic analyses of each particular L1 sub-lineage not only revealed varying patterns of inter-regional spread and population dynamics, but also connected the dots between the virus’s evolutionary history, historical PRRS outbreaks, and potential factors facilitating disease spread. Apart from the first emergence caused by the primitive L1 viruses (L1D), L1F was the earliest well-described sub-lineage and contributed to the second wave of the effective population size of L1, which peaked in the mid-2000s. A virus belonging to this sub-lineage was first isolated from a sample collected in 2001 from a severe PRRS case in southern Minnesota; this isolate is widely referred to as MN184 [80] according to the 1-8-4 ORF5 restriction fragment length polymorphism (RFLP) pattern [66]. L1F viruses were mainly detected in Midwestern regions, with multiple introductions to the Southwest [63]. Interstate swine movement records in 2001 further supported this finding, showing that the Southwest was the second-most frequent destination for pig movements from the Upper Midwest [76]. Currently, L1F appears almost extinct in the U.S., with fewer than two sequences detected each year since 2019 (Figure 3).

Between 2007 and 2008, two virulent PRRSV-2 strains that were responsible for regional outbreaks in Iowa and Minnesota were isolated. They were initially designated as 1-18-2 [81] and NADC-30 [82] strains, which were eventually classified as part of the sub-lineages L1BG and L1C, respectively. These coincided with the third wave of L1 population expansion in the early 2010s, which was influenced by the rise of those sub-lineages along with the sub-lineage L1F. Unlike the previous wave, the estimated inter-regional spread of L1C and L1BG during this wave was prevalent in the eastern part of the country as well as in the Upper Midwest. Patterns of spread completely shifted during the most recent wave in the late 2010s, to which L1A(2) and L1H were the primary contributors (Supplementary Video S1). L1A(2) was the infamous and virulent 1-7-4 strain first detected in 2014–2015 [83,84]. Our analysis confirmed unpublished reports that its spreading hotspot was the Eastern U.S., where the virus spread widely before spilling out into the Midwest [85]. 

In contrast, L1H (80% of the clade members were RFLP 1-8-4 [18]) appears to be an endemic virus that is mostly confined to the Southwest; we found few published epidemiological and virological characterizations of this particular group of viruses. The impact of recent and widespread outbreaks in the Upper Midwest in 2020–2021, caused by the novel L1C-1-4-4 [86] variant, was not captured by our analysis despite the availability of a number of ORF5 sequences. This may be because the novel L1C variant, as of 2021, was not yet so widely spread at the national scale that it significantly contributed to the virus’s overall population and spatiotemporal dynamics.

The inter-regional long-distance spread of PRRSV-2 has been an unavoidable consequence of the vertical integration of U.S. swine production systems, and L1 viruses have evolved in parallel with the expansion of U.S. swine production. After entering the country around the late 1990s, L1 viruses were almost impossible to contain in the Midwestern U.S. since hog and pig inventories were growing rapidly in new areas of the country, including the eastern state of North Carolina and several southwestern states (Oklahoma, Colorado, Texas, and Utah) during the same period [87]. In 2013, Shi et al. estimated that L1 viruses largely circulated only between Canada and the Midwestern regions (the Lake States, Corn Belt, and Northern Plains); meanwhile, the East (Appalachia) was one of the spreading hotspots for non-L1 lineages (Lineages 5–9) [28]. Over time, with increasing L1 prevalence, we captured the geographical shift in the spread of L1. The Eastern region became another spreading hotspot for particular sub-lineages (L1C and L1A(2)) during the third and the fourth waves of L1, likely because it is now the second largest swine producer after the Midwest [43] and the number of outgoing animal shipments is comparable to or sometimes higher than that in the Midwest [88]. These spatial dynamics of the spread may not be exclusive to PRRSV-2 but may be applicable to other swine diseases. Swine influenza, for example, appears to have been introduced into the Midwest from Canada, and new spreading hotspots in the East or other regions are also apparent in phylogeographic analyses of swine influenza in the U.S. [78,79]. Such a correspondence indicates that the dissemination patterns of multiple pathogens in the U.S. swine industry have common determinants, which are likely related to the structural and demographic organization of the industry. 

The U.S. swine industry has been characterized by multi-site pig production for several decades [89,90] and animal movement is a key component that keeps the production flow uninterrupted [91]. The long-distance transport of live animals, cull hogs [92], feed, personnel, and equipment is driven by the uneven distribution of feed resources, production phases, and slaughtering facilities between geographic regions, and/or by contractual relationships between sites [91]. All of these logistical factors are potential risk factors that help to transmit PRRSV-2 via either direct or indirect contacts [20]. In addition, variation in protective measures, such as biosecurity practices and vaccinations, may cause unpredictable changes to the patterns of viral spread, though we were not able to assess these here. Further information relating to field samples, such as the production type of an infected farm, the immunization status, and farm-related transportations before an outbreak, could be useful factors to include in phylogeographic regressions; they can be employed within the DTA framework [93,94] to estimate factors associated with inter-regional transmission. The fraction of sequenced samples relative to the number of actual cases could improve sub-sampling techniques and prevent the occurrence of biases related to unequal sampling/sequencing efforts between regions [62].

We found no support for the hypothesis that selection pressures varied spatially. Episodic diversifying selection along the L1 virus phylogenetic tree was rarely detected. The few branches with evidence of episodic selection were related neither to the (re-)emergence of L1 sub-lineages nor to the expansion of any virus variant. This is not surprising, as such analyses are typically used to assess a virus’s adaptation to a different host species [95,96,97,98,99,100]. Accordingly, we concluded that there is little evidence for episodic selection in PRRSV-2 L1’s evolutionary population dynamics. The estimation of episodic selection takes into account overall genetic changes within ORF5 across each branch of the tree. Since many parts of ORF5 are strongly conserved, using this branch-site model potentially obscures positive selection that occurs in specific amino acid residues, e.g., antigenically important amino acid sites that have been detected using site-specific models [3,101].

## 5. Conclusions

Here, we used phylogeographic models to analyze the spread of PRRSV-2 L1 in the U.S. over 30 years, covering the time period from the first appearance of L1 in the U.S. to its current dominance. Canada was estimated to be the most likely origin of the virus, which was introduced to the U.S. in the late 1990s, potentially through imports of feeder and finisher animals. Since then, the virus has diverged into several sub-lineages, each displaying different spatiotemporal dynamics. Once L1 viruses were established in the U.S., the swine-dense Upper Midwest region became a major hotspot for inter-regional spread. The contribution of the Eastern region of the U.S. to inter-regional spread has increased in the last decade, potentially due to the expansion of hog inventories within that region. The national-scale overview of recent PRRSV spatiotemporal dynamics presented here provides a contextual framework to aid in the design of PRRS prevention and control strategies for pig farming and disease control.

## Data Availability

The data used for the analyses in this study may be made available upon request to the corresponding author (K.V.). Only some of the data are publicly available (the NCBI GenBank sequencing data), as most PRRSV-2 genetic sequences are confidential diagnostic data obtained from third parties (i.e., the participating swine production system and veterinarians who submitted samples for diagnosis to one of the universities’ VDLs).

## Supplementary Materials

The following supporting information can be downloaded at: https://www.mdpi.com/article/10.3390/pathogens12050740/s1, Figure S1: ORF5 sequences gathering and filtering process.; Figure S2: Geographical distribution of pig farming (left) and ORF5 sequences from the full dataset (right) by region (top) and state (bottom).; Figure S3: Geographical distribution of ORF5 sequences from each subsampled dataset.; Figure S4: Temporal distribution of ORF5 sequences from each subsampled dataset colored by source, sampling region, and pre-identified sub-lineage.; Figure S5: Probability of region of origin (top) and Median tMRCA with 95% HPD interval (bottom) of each L1 sub-lineage and overall L1 from all runs compared between different subsampling techniques.; Figure S6: Probability of region of origin (top) and Median tMRCA with 95% HPD interval (bottom) of each L1 sub-lineage and overall L1 from all runs compared between different phylogeographic approaches.; Figure S7: Key results from TreeTime analysis on the full L1 dataset. (A) The time-scaled phylogenetic tree with tip colored by sampling region and internal branch colored by inferred ancestral region. (B) Probability (of region of origin of each L1 sub-lineage and overall L1. (C) The similar timed tree with tip colored by classified sub-lineage. (D) Median tMRCA with 95% HPD interval of L1 and its sub-lineages.; Figure S8: Comparison of inter-regional spread of PRRSV-2 L1 in the U.S. between different phylogeographic analyses and subsampling techniques in map and arrows format.; Figure S9: Comparison of inter-regional spread of PRRSV-2 L1 in the U.S. between different phylogeographic analyses and subsampling techniques in matrix format.; Figure S10: Median between-region transitions of each L1 sub-lineage estimated by DTA on spatio-temporal stratified sampled sets in matrix format.; Figure S11: Proportion of ancestral region (trait of branch) through time between different phylogeographic analyses and subsampling techniques.; Figure S12: Proportion of the region receiving the virus from another region (regional transition) through time between different phylogeographic analyses and subsampling techniques.; Figure S13: Comparison of L1 population dynamics estimated by Bayesian Skygrid analysis on datasets from different subsampling techniques. Thin lines in the background are median effective population size of each run. Thick lines with bands are LOESS smoothing curve with 95% probability interval of the median population sizes from five runs of each subsampling technique. Color of line and band on the plot represents subsampling technique.; Figure S14: Comparison of branch median substitution rate between sub-lineages (left) and temporal distribution of the rate between sub-lineages (right).; Figure S15: Comparison of branch median substitution rate between regions (left) and temporal distribution of the rate between regions (right).; Figure S16: Comparison of branch specific dN/dS ratio through time between sub-lineages (left) and between regions (right). Black circle locates a significant episodic positive selected branch.; Figure S17: Comparison of branch specific dN/dS ratio (y-axis) and substitution rate (x-axis) between sub-lineages (left) and between regions (right). Black circle locates a significant episodic positive selected branch.; Table S1: Designated geographic regions, pig inventory, and available PRRSV-2 L1 ORF5 sequences.; Table S2: Parameter settings in different phylogeographic approaches.; Video S1: Inter-regional spread of PRRSV-2 L1 in the U.S. through time inferred from the full L1 dataset (n = 19,395) using TreeTime in the Nextstrain platform.
