# Supplementary material for: Mapping the Dynamics of Contemporary PRRSV-2 Evolution and Its Emergence and Spreading Hotspots in the U.S. Using Phylogeography
Source: Pathogens. 2023 May 20;12(5):740. doi: 10.3390/pathogens12050740 (PMC10222675; doi:10.3390/pathogens12050740)
Supplement: Supplementary file 1 [file pathogens-12-00740-s001.zip › pathogens-2397113-supplementary.pdf]

Supplementary data

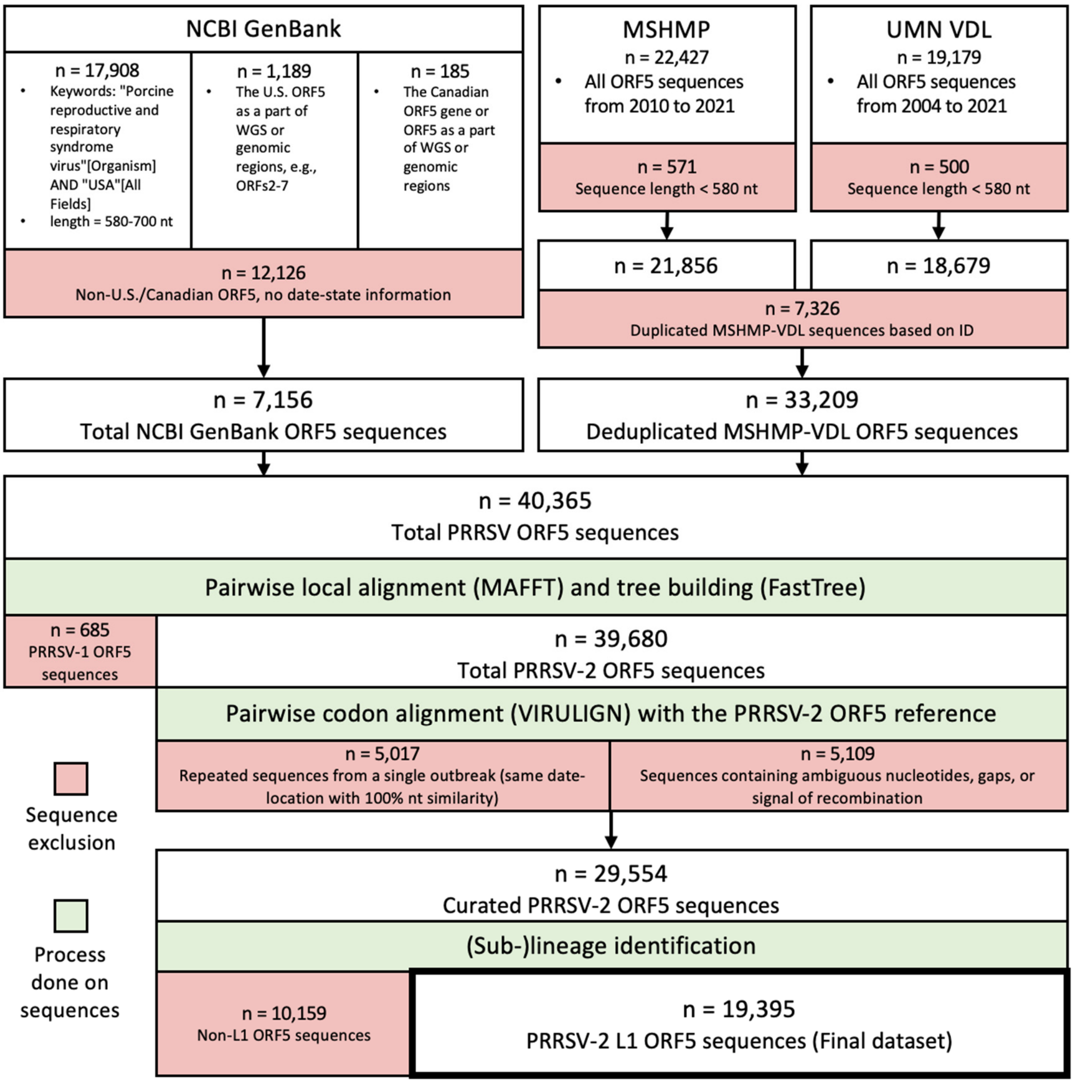

Supplementary Figure S1. ORF5 sequences gathering and filtering process.

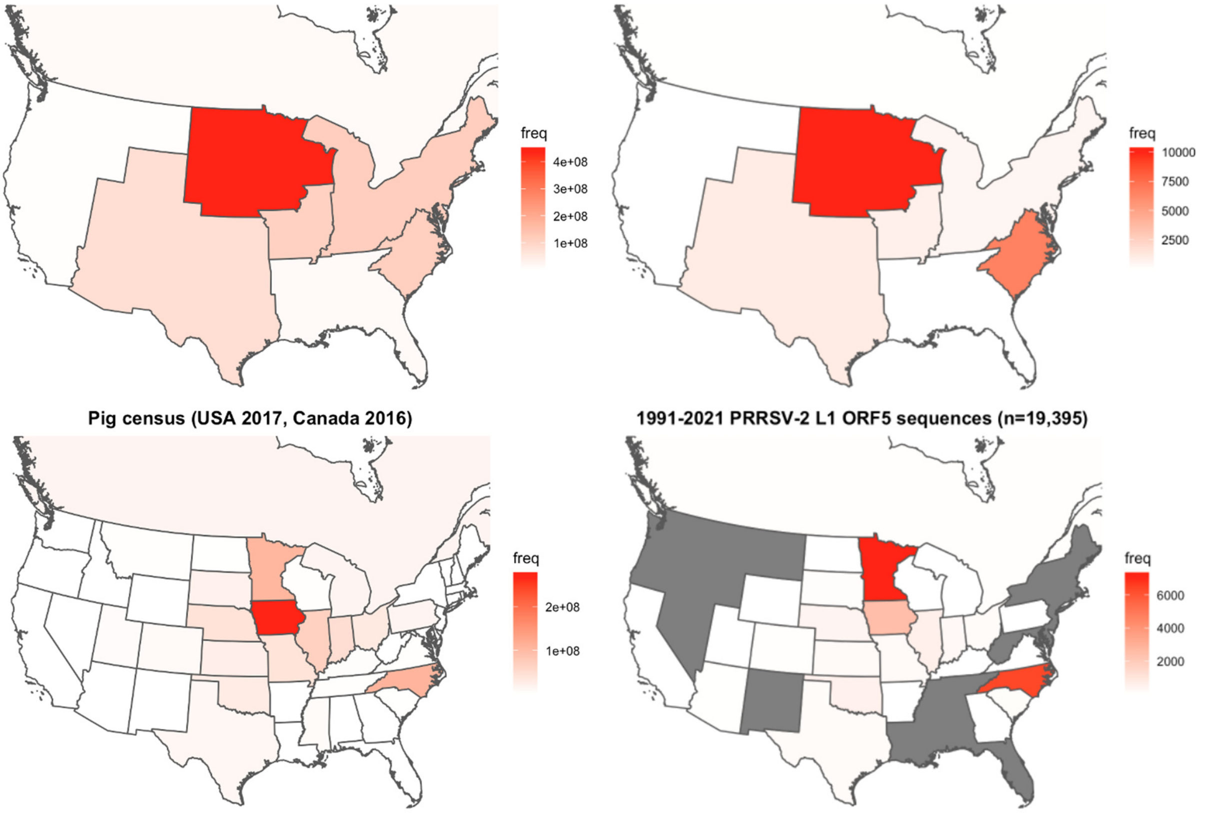

Supplementary Figure S2. Geographical distribution of pig farming (left) and ORF5 sequences from the full dataset (right) by region (top) and state (bottom).

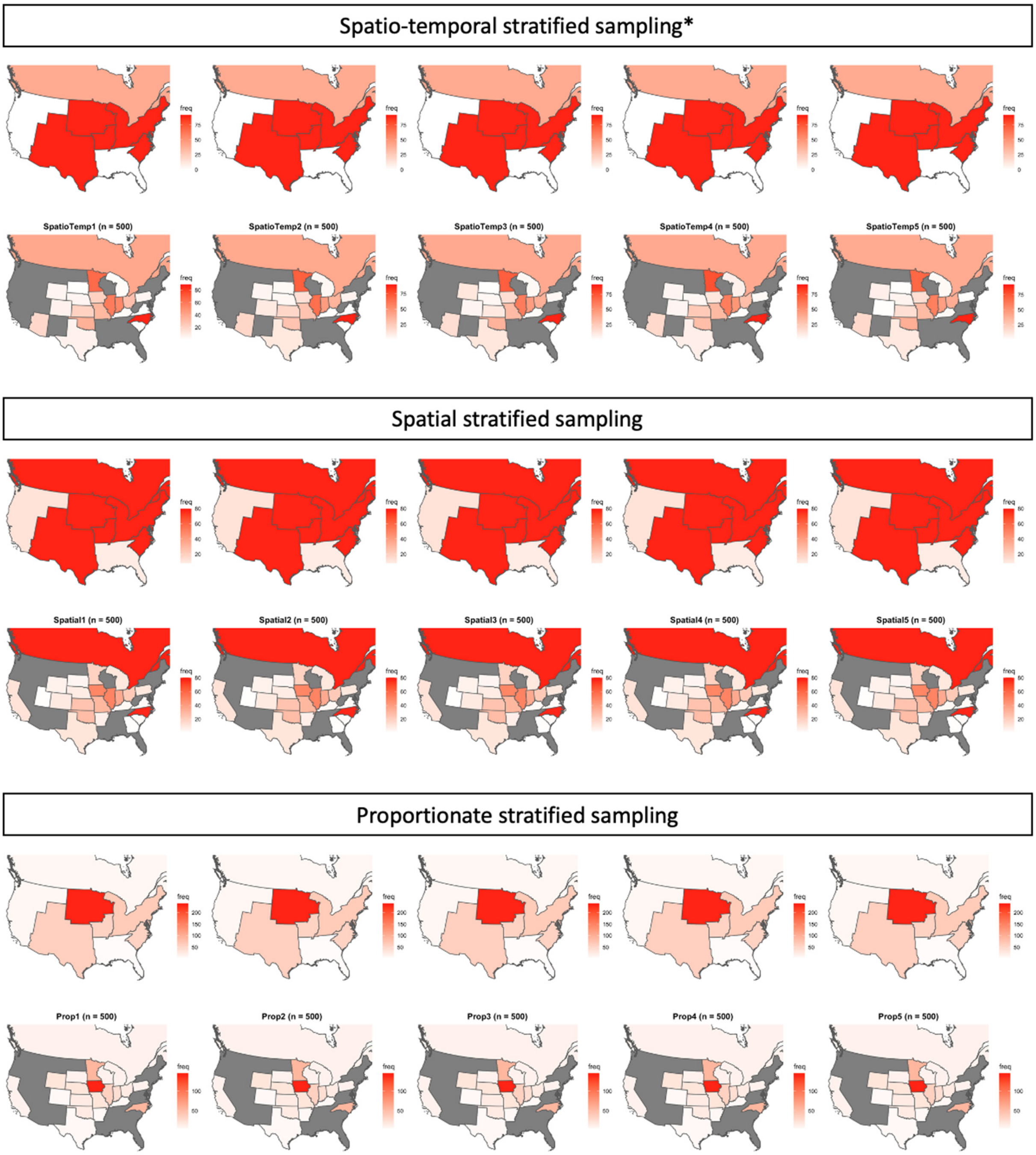

Supplementary Figure S3. Geographical distribution of ORF5 sequences from each subsampled dataset.

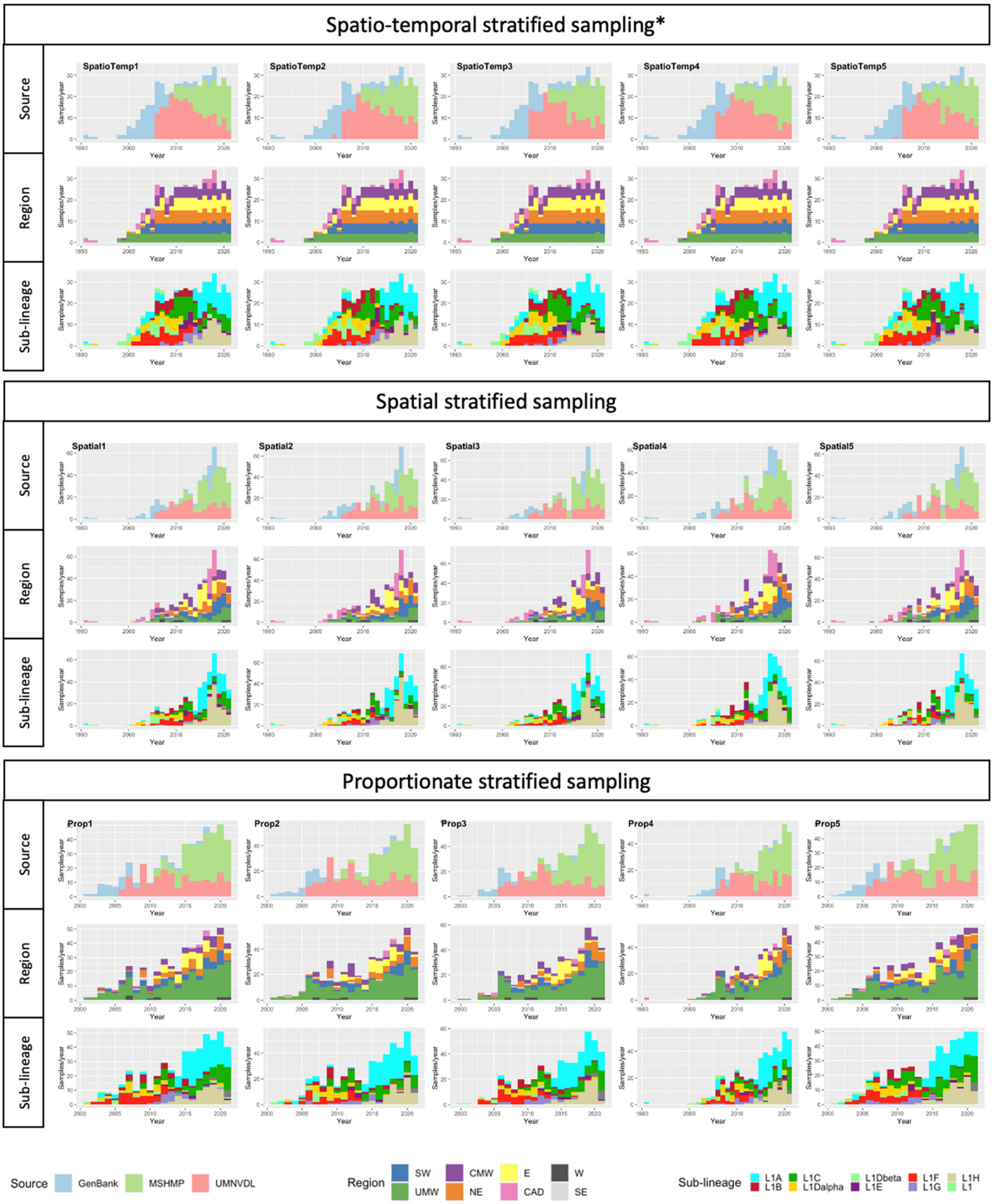

Supplementary Figure S4. Temporal distribution of ORF5 sequences from each subsampled dataset colored by source, sampling region, and pre-identified sub-lineage.

Discrete trait analysis (DTA) in BEAST v1.10.4		
Spatio-temporal stratified sampling*	Spatial stratified sampling	Proportionate stratified sampling

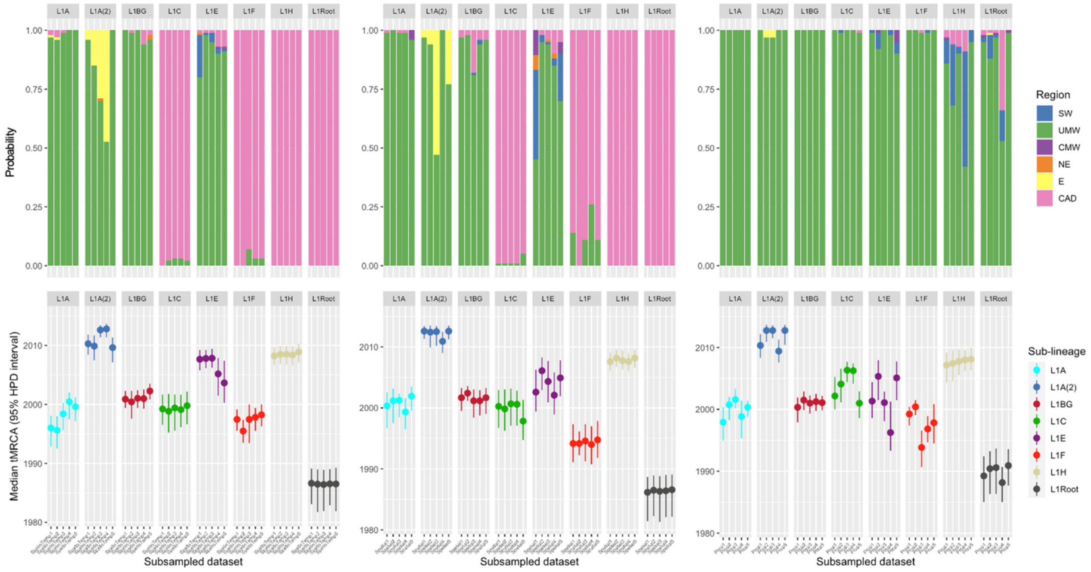

Supplementary Figure S5. Probability of region of origin (top) and Median tMRCA with 95% HPD interval (bottom) of each L1 sub-lineage and overall L1 from all runs compared between different subsampling techniques.

Spatio-temporal stratified sampling		
DTA (BEAST 1)*	TreeTime (Nextstrain)	MASCOT (BEAST 2)

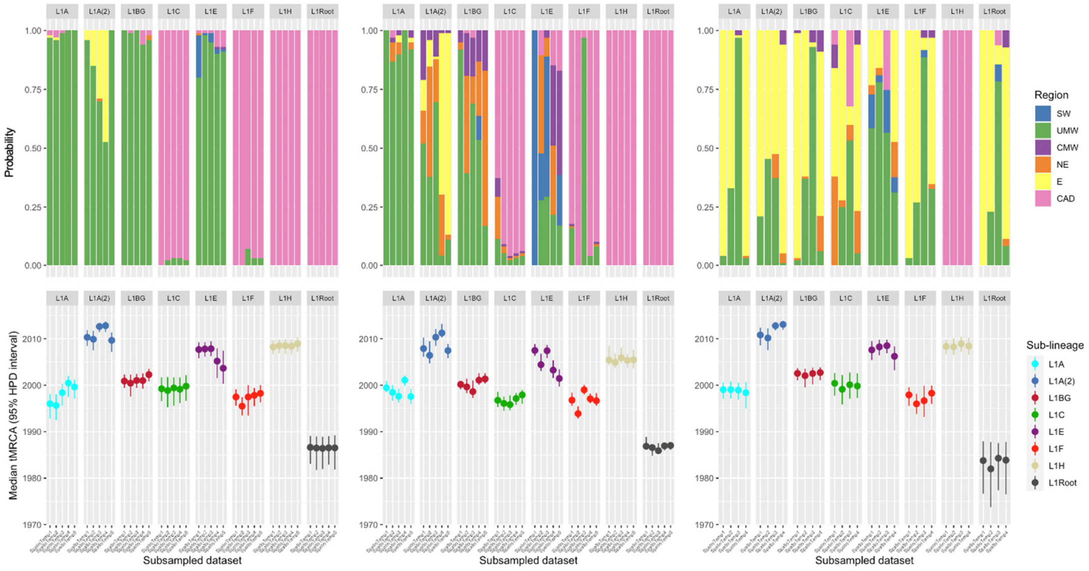

Supplementary Figure S6. Probability of region of origin (top) and Median tMRCA with 95% HPD interval (bottom) of each L1 sub-lineage and overall L1 from all runs compared between different phylogeographic approaches.

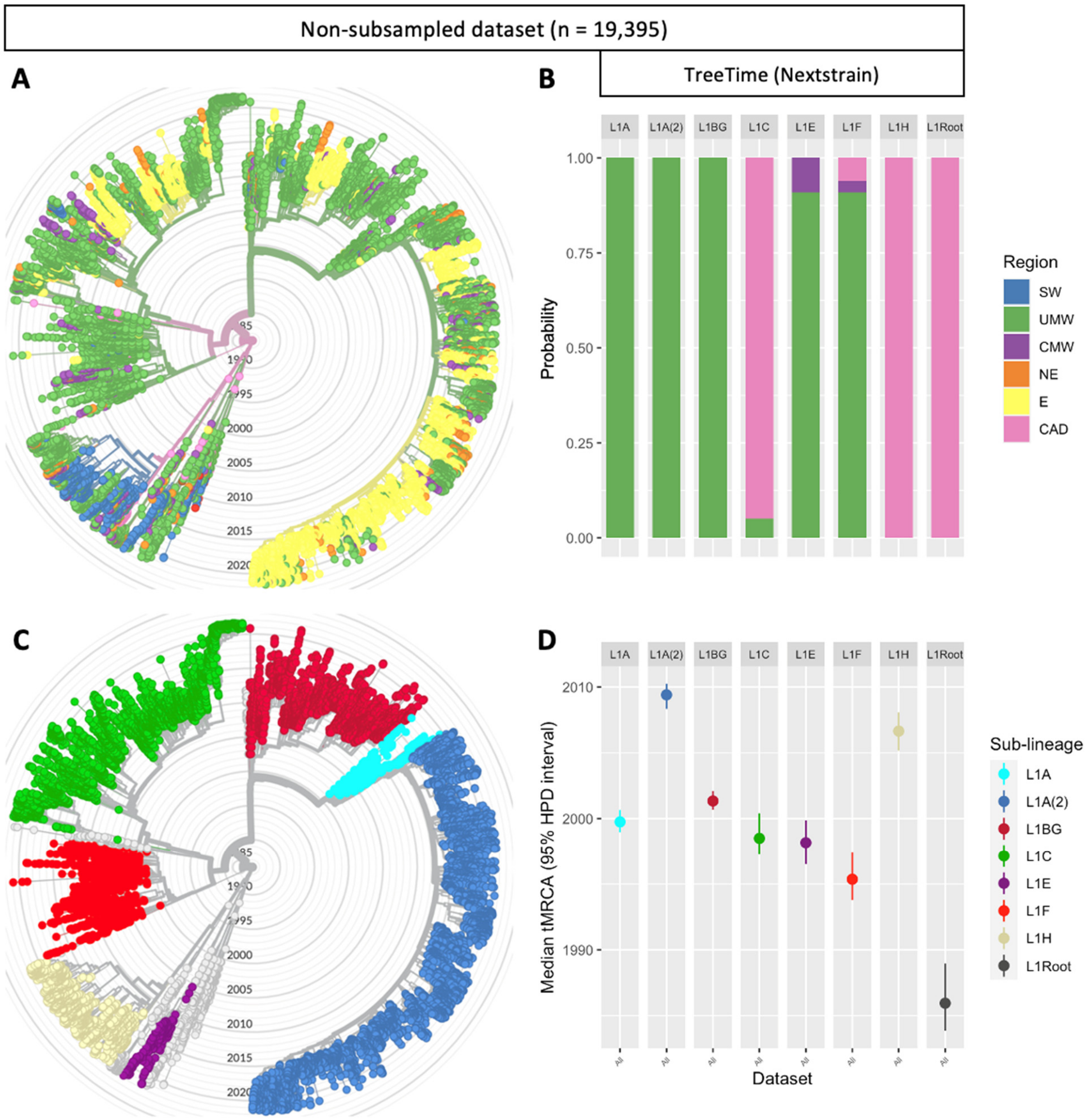

Supplementary Figure S7. Key results from TreeTime analysis on the full L1 dataset. (A) The time-scaled phylogenetic tree with tip colored by sampling region and internal branch colored by inferred ancestral region. (B) Probability (of region of origin of each L1 sub-lineage and overall L1. (C) The similar timed tree with tip colored by classified sub-lineage. (D) Median tMRCA with 95% HPD interval of L1 and its sub-lineages.

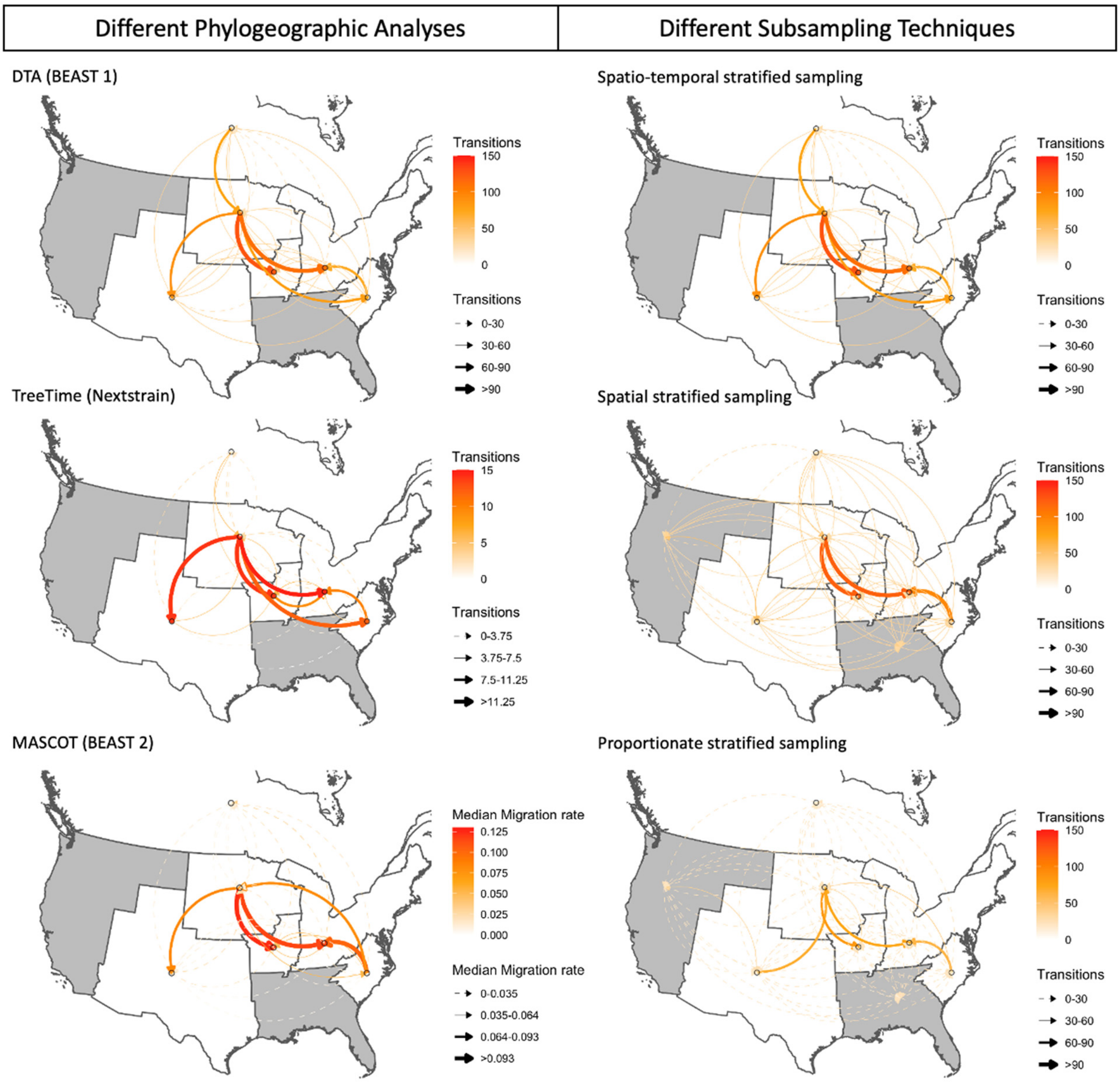

Supplementary Figure S8. Comparison of inter-regional spread of PRRSV-2 L1 in the U.S. between different phylogeographic analyses and subsampling techniques in map and arrows format.

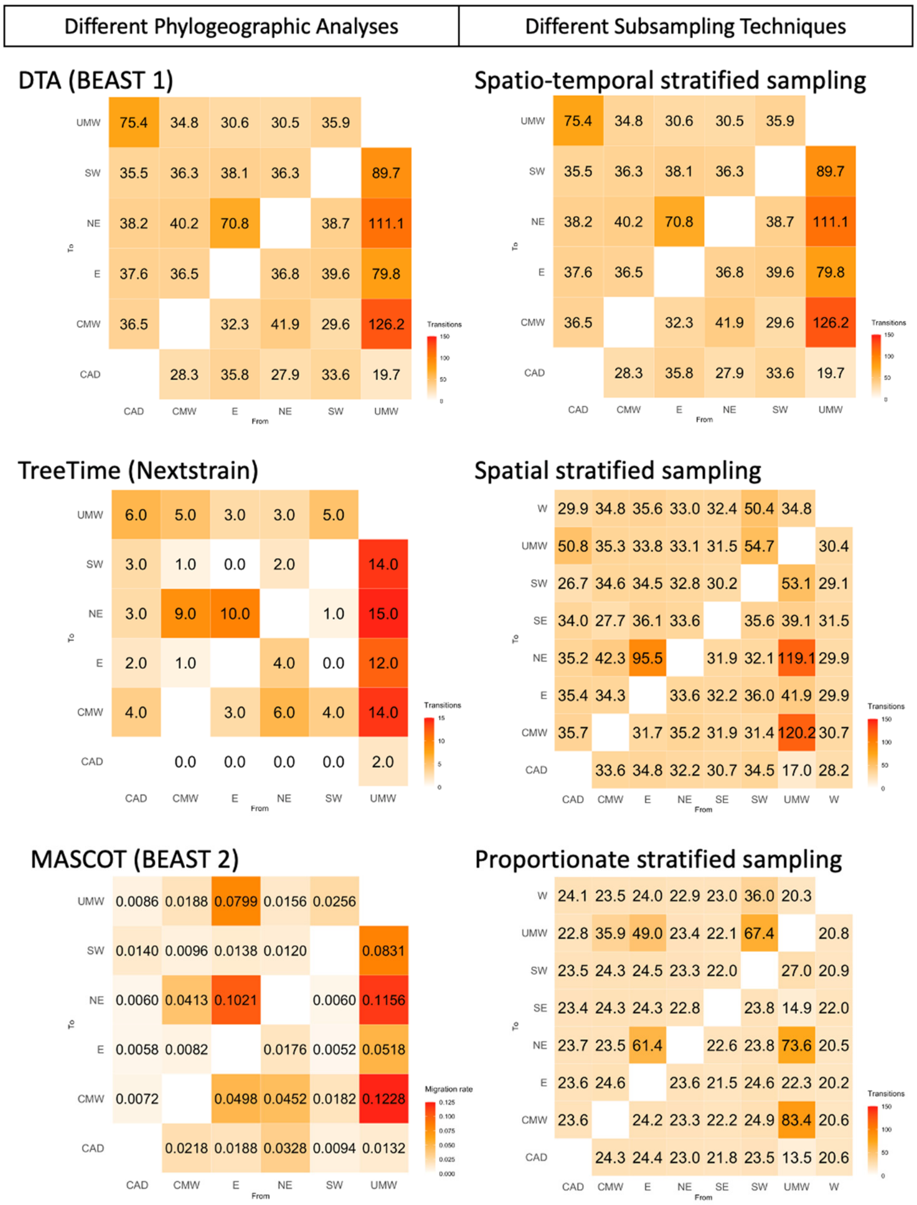

Supplementary Figure S9. Comparison of inter-regional spread of PRRSV-2 L1 in the U.S. between different phylogeographic analyses and subsampling techniques in matrix format.

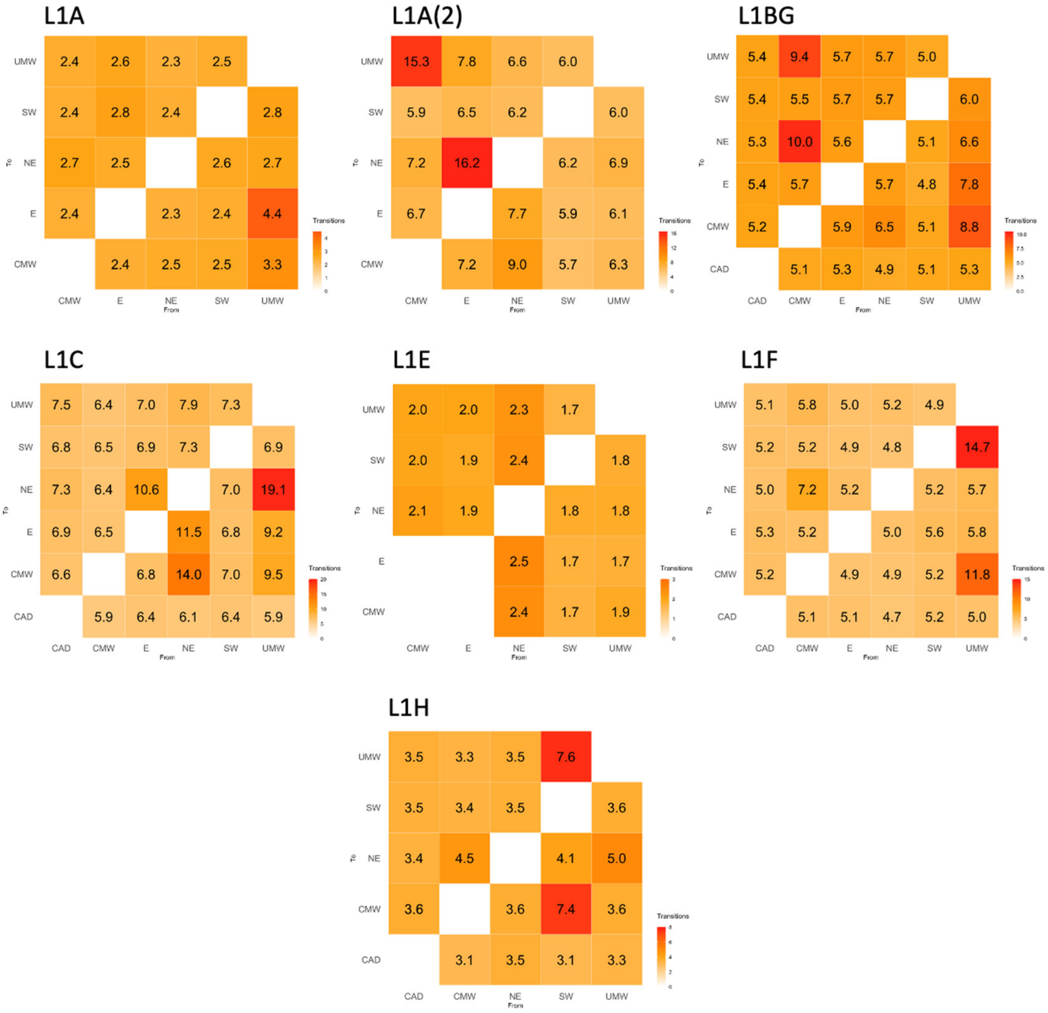

Supplementary Figure S10. Median between-region transitions of each L1 sub-lineage estimated by DTA on spatio-temporal stratified sampled sets in matrix format.

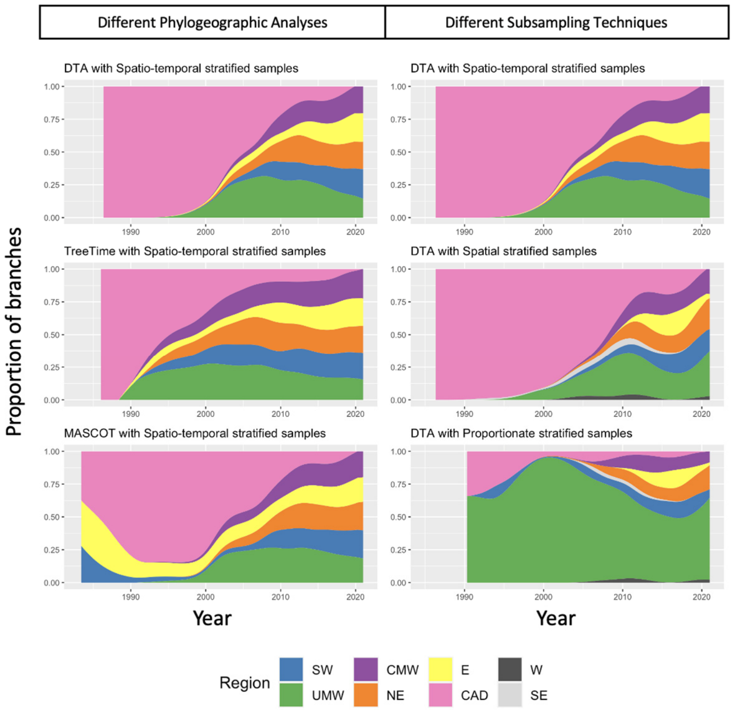

Supplementary Figure S11. Proportion of ancestral region (trait of branch) through time between different phylogeographic analyses and subsampling techniques.

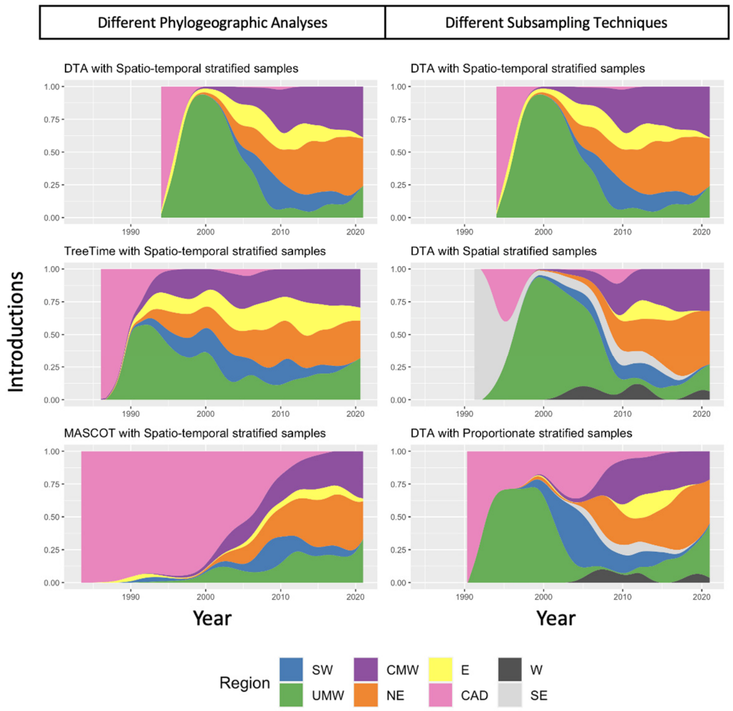

Supplementary Figure S12. Proportion of the region receiving the virus from another region (regional transition) through time between different phylogeographic analyses and subsampling techniques.

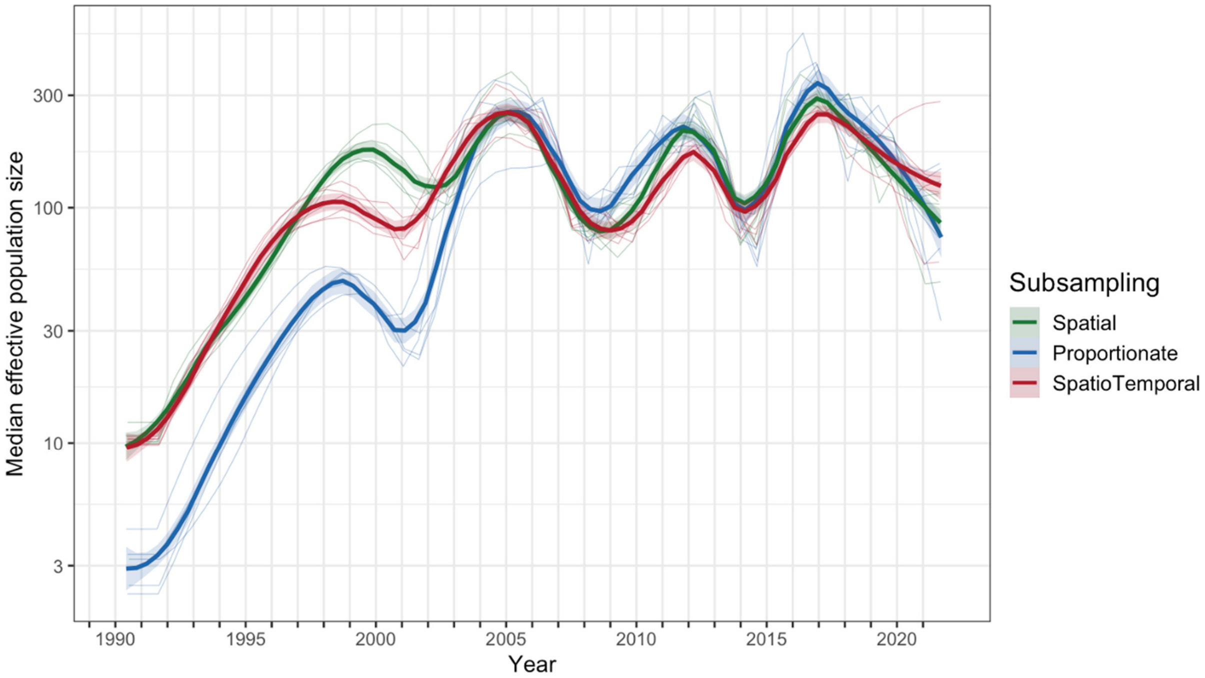

Supplementary Figure S13. Comparison of L1 population dynamics estimated by Bayesian Skygrid analysis on datasets from different subsampling techniques. Thin lines in the background are median effective population size of each run. Thick lines with bands are LOESS smoothing curve with 95% probability interval of the median population sizes from five runs of each subsampling technique. Color of line and band on the plot represents subsampling technique.

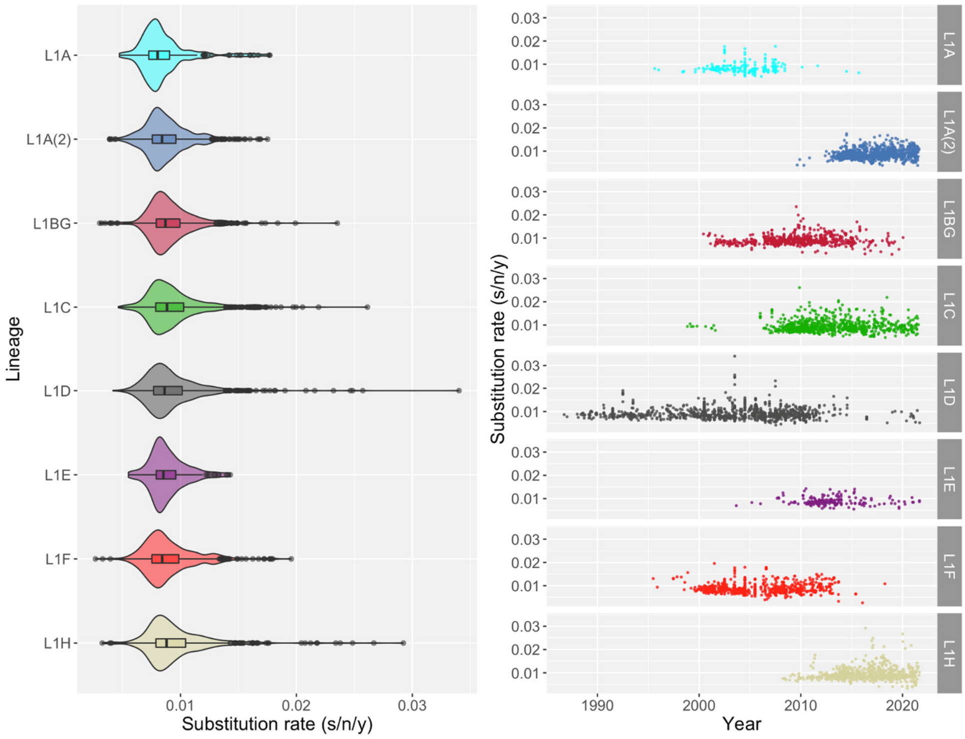

Supplementary Figure S14. Comparison of branch median substitution rate between sub-lineages (left) and temporal distribution of the rate between sub-lineages (right).

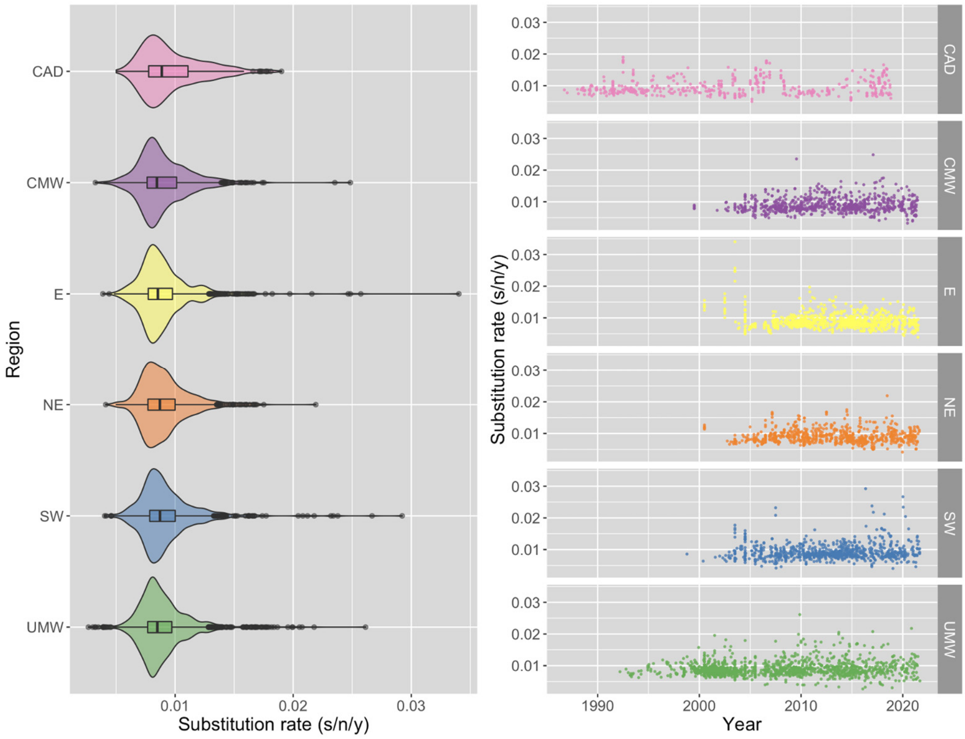

Supplementary Figure S15. Comparison of branch median substitution rate between regions (left) and temporal distribution of the rate between regions (right).

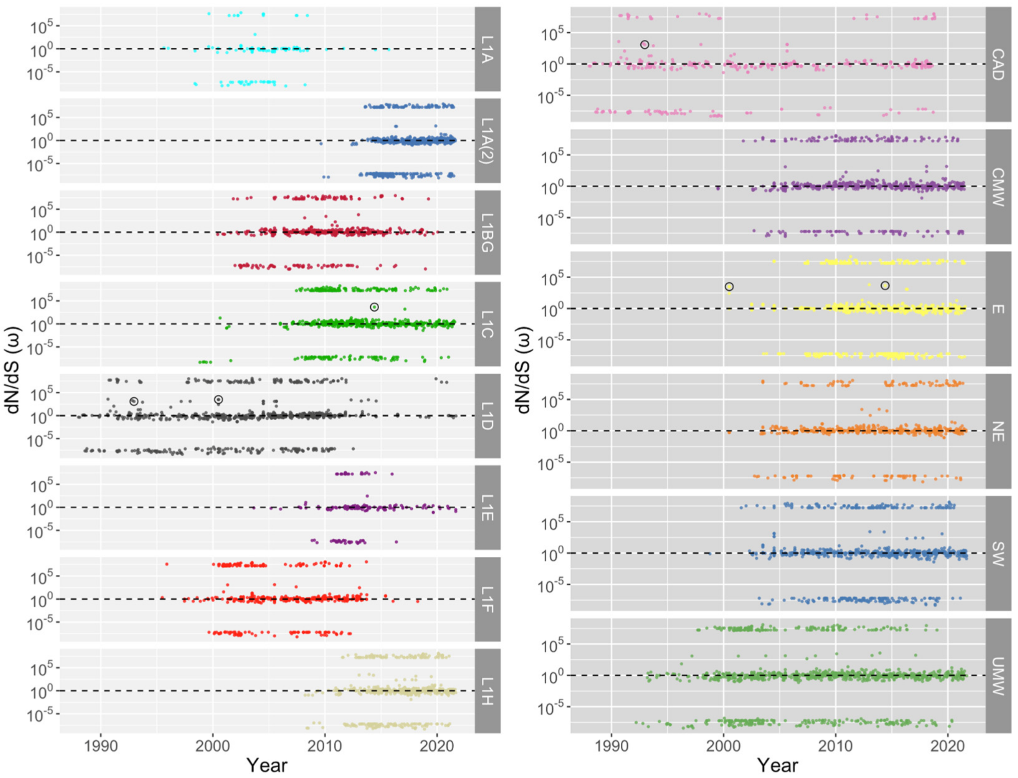

Supplementary Figure S16. Comparison of branch specific dN/dS ratio through time between sub-lineages (left) and between regions (right). Black circle locates a significant episodic positive selected branch.

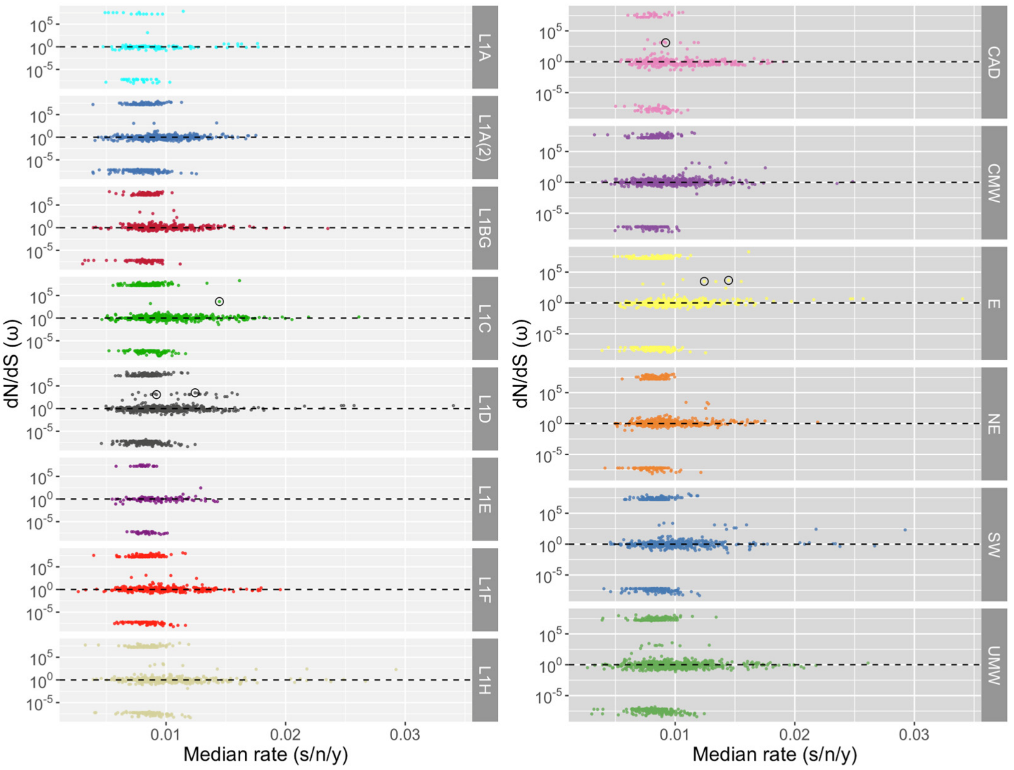

Supplementary Figure S17. Comparison of branch specific dN/dS ratio (y-axis) and substitution rate (x-axis) between sub-lineages (left) and between regions (right). Black circle locates a significant episodic positive selected branch.

Supplementary Table S1. Designated geographic regions, pig inventory, and available PRRSV-2 L1 ORF5 sequences.

Region	State	2017 US pig inventory (heads)	2017 US pig inventory (%)	Available L1 ORF5 (sequences)	Available L1 ORF5 sequences (%)
**Southwest**	Arizona	27,692	0.003	46	0.238
**Southwest**	Utah	5,504,403	0.624	1	0.005
**Southwest**	Wyoming	1,063,747	0.121	24	0.124
**Southwest**	Colorado	8,479,673	0.961	76	0.393
**Southwest**	New Mexico	18,346	0.002	0	0.000
**Southwest**	Kansas	24,541,273	2.781	226	1.170
**Southwest**	Oklahoma	27,403,245	3.106	525	2.718
**Southwest**	Texas	11,221,159	1.272	159	0.823
**Upper Midwest**	North Dakota	1,971,079	0.223	1	0.005
**Upper Midwest**	South Dakota	19,998,670	2.266	103	0.533
**Upper Midwest**	Nebraska	42,995,739	4.873	423	2.190

**Upper Midwest**	Minnesota	104,954,069	11.894	7,356	38.084
**Upper Midwest**	Iowa	278,658,346	31.580	2,490	12.892
**Upper Midwest**	Wisconsin	3,354,146	0.380	11	0.057
**Central Midwest**	Missouri	40,698,393	4.612	234	1.211
**Central Midwest**	Illinois	66,899,427	7.582	593	3.070
**Northeast**	Michigan	15,096,398	1.711	12	0.062
**Northeast**	Indiana	49,709,257	5.634	260	1.346
**Northeast**	Kentucky	4,737,803	0.537	6	0.031
**Northeast**	Ohio	31,232,937	3.540	161	0.834
**Northeast**	West Virginia	46,973	0.005	0	0.000
**Northeast**	Maryland	162,713	0.018	0	0.000
**Northeast**	Delaware	46,898	0.005	0	0.000
**Northeast**	Pennsylvania	14,747,532	1.671	34	0.176
**Northeast**	New Jersey	76,678	0.009	0	0.000
**Northeast**	New York	489,548	0.055	0	0.000
**Northeast**	Connecticut	30,037	0.003	0	0.000
**Northeast**	Rhode Island	15,004	0.002	0	0.000
**Northeast**	Vermont	41,779	0.005	0	0.000
**Northeast**	New Hampshire	27,026	0.003	0	0.000
**Northeast**	Massachusetts	37,094	0.004	0	0.000
**Northeast**	Maine	39,058	0.004	0	0.000
**East**	Virginia	3,192,298	0.362	33	0.171
**East**	North Carolina	107,021,897	12.129	6,389	33.078
**East**	South Carolina	2,068,421	0.234	132	0.683
**West**	Washington	139,245	0.016	0	0.000
**West**	Oregon	99,375	0.011	0	0.000
**West**	California	826,252	0.094	12	0.062
**West**	Nevada	5,510	0.001	0	0.000
**West**	Idaho	167,762	0.019	0	0.000
**West**	Montana	2,366,551	0.268	0	0.000
**Southeast**	Arkansas	1,453,385	0.165	7	0.036
**Southeast**	Louisiana	53,068	0.006	0	0.000
**Southeast**	Tennessee	2,661,681	0.302	0	0.000
**Southeast**	Mississippi	6,416,420	0.727	0	0.000
**Southeast**	Alabama	521,985	0.059	0	0.000
**Southeast**	Georgia	929,804	0.105	1	0.005
**Southeast**	Florida	130,663	0.015	0	0.000
**Canada**	NA	14,091,503 (2016)	NA	80	NA

Supplementary Table S2. Parameter settings in different phylogeographic approaches.

Software	BEAST v1.10.4	TreeTime v0.8.5	BEAST v2.5.1
**Datasets used**	All subsampled datasets (n = 15, 500 seqs each)	Spatio-temporal subsampled datasets (n = 5, 500 seqs each) and non-subsampled dataset (19,395 seqs)	Spatio-temporal subsampled datasets (n = 5*, 500 seqs each)
**Nucleotide substitution model**	GTR + I + G	GTR	GTR + G
**Molecular clock model**	Uncorrelated relaxed clock, Log-normal distribution	Strict clock	Uncorrelated relaxed clock, Log-normal distribution
**Model for phylogeography**	Non-reversible CTMC (DTA)	GTR (mugration model)	Marginal approximation of the structured coalescent (MASCOT)
**Coalescent model (population dynamics)**	Bayesian GMRF Skygrid	Constant coalescent rate	Structured coalescent with constant effective population size and migration rate
**MCMC chain length**	300 million	No MCMC running	300 million

*Only 4 out of 5 datasets could be accomplished in BEAST 2 without error. (Potential errors: https://taming-the-beast.org/tutorials/Mascot-Tutorial/
